# Evaluation of the impact of renal impairment on the pharmacokinetics of glasdegib in otherwise healthy volunteers

**DOI:** 10.1007/s00280-020-04207-9

**Published:** 2021-01-03

**Authors:** Naveed Shaik, Robert R. LaBadie, Brian Hee, Geoffrey Chan

**Affiliations:** 1 Clinical Pharmacology, Pfizer Inc, La Jolla, CA USA; 2 Clinical Statistics, Pfizer Inc, Groton, CT USA; 3 Clinical Development Oncology, Pfizer Inc, Collegeville, PA USA

**Keywords:** Glasdegib, Pharmacokinetics, Renal impairment, Safety

## Abstract

**Purpose:**

Glasdegib is being developed for indications in myeloid malignancies. The effect of renal impairment on the pharmacokinetics (PK) of a single, oral, 100-mg glasdegib dose under fasted conditions was assessed.

**Methods:**

Open-label, parallel-group study (NCT03596567). Participants of good general health were selected and categorized, based on their estimated glomerular filtration rate, into normal (≥ 90 mL/min), moderate (≥ 30 to < 60 mL/min), or severe (< 30 mL/min) renal impairment groups. Blood samples were collected up to 120 h post-dose. PK exposure parameters were calculated using non-compartmental analysis.

**Results:**

All 18 participants completed the study. Respectively, ratios of adjusted geometric means (90% confidence interval) for glasdegib area under the curve from time 0 to infinity and peak plasma concentration versus normal participants were 205% (142–295%) and 137% (97–193%) in the moderate group, and 202% (146–281%) and 120% (77–188%) in the severe group. Glasdegib median time to peak plasma concentration was 2.0 h in both impairment groups and 1.5 h in the normal group. Mean oral clearance was decreased by approximately 50% in both renal impairment groups compared with the normal group. The plasma-free fraction of glasdegib was not altered by renal impairment. Five all-causality adverse events were reported in three participants; two were considered treatment-related.

**Conclusion:**

The similar changes in exposure observed for participants with renal impairment, coupled with the known safety data from clinical experience, suggest that a lower starting dose of glasdegib may not be required for moderate or severe renal impairment.

**Trial registration:** ClinicalTrials.gov: NCT03596567 (started May 17, 2018).

**Electronic supplementary material:**

The online version of this article (10.1007/s00280-020-04207-9) contains supplementary material, which is available to authorized users.

## Introduction

The Hedgehog (Hh) signaling pathway regulates cell differentiation and embryogenesis, and is typically silenced in adult tissues. Aberrant Hh signaling has been identified in a variety of human leukemia types and leukemia stem cells [1–5]. Glasdegib binds to and inhibits Smoothened, a transmembrane protein involved in Hh signal transduction [6].

Glasdegib 100 mg once daily (QD) in combination with low-dose cytarabine is approved by the US Food and Drug Administration for treating patients with newly diagnosed acute myeloid leukemia (AML) who are ≥ 75 years old or who have comorbidities that preclude use of intensive induction chemotherapy. The combination was also previously granted orphan designation by the European Medicines Agency (EMA) [7, 8] and has recently received initial authorization by the EMA for use in combination with low-dose cytarabine, for the treatment of newly diagnosed de novo or secondary AML in adult patients who are not candidates for standard induction chemotherapy [9].

In a Phase I pharmacokinetics (PK) and safety study, glasdegib exhibited dose-proportional PK, and the maximum tolerated dose (MTD) of glasdegib monotherapy was established as 400 mg QD [10]. Clinical trials of glasdegib as a combination therapy are underway for indications in myeloid malignancies (AML and myelodysplastic syndromes [MDS]).

Glasdegib is primarily metabolized by cytochrome P450 (CYP)3A4/5, and glasdegib and its metabolites are primarily eliminated via urine and feces [11]. Approximately 16.5% and 19.5% of glasdegib were excreted unchanged in urine and feces, respectively, and corresponding dose recovery was 48.9% and 41.7% [11]. Consistent with this, in a drug–drug interaction (DDI) study, administration of glasdegib with a strong CYP3A4 inhibitor, ketoconazole, led to a 140% increase in geometric mean area under the plasma concentration–time curve (AUC) from time 0 to infinity (AUC_∞_) and a 40% increase in geometric mean maximum plasma concentration (C_max_) [12]. Based on the magnitude of the DDI and the target population of patients with AML, who routinely require use of anti-fungal agents (such as azoles, which are CYP3A4 inhibitors), the clinical dose of glasdegib was selected as 100-mg QD for further clinical evaluation.

A population PK analysis of patients with hematologic and solid tumors demonstrated that baseline creatinine clearance (CrCl; based on Cockcroft-Gault equation) was a statistically significant predictor of variability in glasdegib clearance [13]. However, glasdegib clearance was similar between patients with mild renal impairment and those with normal renal function, while a 26% decrease in apparent clearance of total drug from plasma (CL/F) was estimated for patients with moderate renal impairment. Renal impairment was defined using the Kidney Disease Outcomes Quality Initiative classification. No data were available in patients with severe renal impairment [13]. Patients with mild renal impairment had similar median weight-normalized glasdegib CL/F relative to patients with normal renal function—6.2 L/h versus 6.5 L/h, respectively. An increase in glasdegib exposure was not expected with mild renal impairment, and the current study would pursue a study design with only normal, moderate, and severe renal impairment groups. Therefore, the aim of this study was to estimate the effect of moderate and severe renal impairment on the PK and safety of a single, oral, 100-mg glasdegib dose.

## Methods

### Study design

This was an open-label, parallel-group study (ClinicalTrials.gov NCT03596567). Participants from the impaired groups were recruited first, and demographics were pooled across study sites and levels of impairment to determine an average value for age and weight. Participants with normal renal function were then recruited so that each participant’s age was within 10 years and weight within 15 kg of the mean of the pooled impaired groups.

Renal function was estimated using both Cockcroft–Gault and Modification of Diet in Renal Disease (MDRD) equations: estimated glomerular filtration rate (eGFR; mL/min/1.73 m^2^) was obtained directly from the laboratory or calculated using the following equation from the MDRD study: 175 × (_Scr,std_)^−1.154^ × (Age)^−0.203^ × (0.742 if female) × (1.212 if African American). Scr,std denotes serum creatinine measured with a standardized assay for serum creatinine. In terms of clearance of renally filtrated drugs, renal elimination capacity was related to absolute GFR. To use the MDRD-derived, body surface area (BSA)-adjusted value of eGFR to obtain absolute GFR (mL/min) for participant assignment to moderate or severe renal impairment groups, this value was multiplied by the individual participant’s BSA (i.e., measured BSA/1.73 m^2^). CrCl was also estimated from a spot serum creatinine measurement using the following Cockcroft–Gault equation: CrCl (mL/min) = [140 – age (years)] × total body weight (kg) × (0.85 for females)/72 × serum creatinine (mg/dL). The eGFR value obtained within 24 h prior to the glasdegib dose was the value used for stratification and group assignment. The CrCl value was recorded at the same time eGFR was determined.

Participants were required to have stable renal function (two serum creatinine values within 20% of each other obtained within a 2-week period) during the screening period. The eGFR value obtained within 24 h prior to the glasdegib dose was the value used for participant stratification and group assignment. The CrCl value was recorded at the same time eGFR was determined.

Although glasdegib can be taken with or without food, the study was performed under fasted conditions, to minimize potentially confounding factors. Following an overnight fast of ≥ 10 h, participants received a single, oral, 100-mg dose of glasdegib with approximately 240 mL of water in the morning on day 1. No food or drink (except water) was permitted for ≥ 4 h post dose. Each participant underwent serial blood samplings to determine plasma concentrations of glasdegib up to 120 h post dose on day 6. PK blood sampling (~ 2 mL) occurred pre-dose, and at 0.5, 1, 2, 4, 6, 10, 24, 48, 72, 96, and 120 h post dose. The eGFR value obtained on day –1 was used for PK analysis. At pre-dose, and at 1 and 2 h post dose, separate blood samples (10 mL) were collected for measurement of protein binding. Physical examinations, electrocardiogram (ECG), vital signs, and clinical laboratory tests were conducted, and adverse events (AEs) were monitored throughout the study. Participants were confined to the unit during the study until completion of PK sampling and safety assessments on the morning of day 6. Participants who withdrew were to be replaced to ensure that six PK-evaluable participants were in each group. A follow-up assessment 28–35 days after the administration of the glasdegib dose was conducted by phone.

The study protocol was approved by an independent institutional review board and was conducted in accordance with the Declaration of Helsinki and Good Clinical Practice guidelines. Informed consent was obtained from all individual participants included in the study.

### Participants

All participants were aged 18–75 years with a body mass index (BMI) of 17.5–40.0 kg/m^2^ and a total body weight > 50.0 kg. Participants had no clinically relevant abnormalities identified by a detailed medical history, full physical examination, 12-lead ECG, or clinical laboratory tests. Participants were excluded if they: had any condition affecting drug absorption; were renal allograft recipients; had urinary incontinence without catheterization; had congenital long QT syndrome, torsades de pointes, or clinically significant ventricular arrhythmias.

Participants with normal renal function had eGFR ≥ 90 mL/min, based on the MDRD equation, and were matched for age (within 10 years), weight (within 15.0 kg), and sex to participants in the impaired renal function groups. Participants were excluded if they: had blood pressure ≥ 140 mm Hg (systolic) or ≥ 90 mm Hg (diastolic); had QT interval > 450 ms or QRS interval > 120 ms.

The following eligibility criteria were applicable for participants with moderate (eGFR ≥ 30 mL/min and < 60 mL/min) or severe (eGFR < 30 mL/min, but not requiring hemodialysis) impairment of renal function: good general health commensurate with the chronic kidney disease population; stable drug regimen, defined as not starting a new drug or changing dosage within 7 days or five half–lives (whichever was longer) before dosing the study drug; any form of renal impairment except acute nephritic syndrome. Good general health was defined as no clinically relevant abnormalities, with the exception of hypertension, diabetes mellitus, hyperparathyroidism, and ischemic heart disease, as long as the participant was medically stable, was on a stable drug regimen, and was able to abide by the fasting conditions of the study. Participants with a history of previous nephritic syndrome, but in remission, were included. Participants were excluded if they: required hemodialysis; had blood pressure ≥ 180 mm Hg (systolic) or ≥ 110 mm Hg (diastolic); had QT interval > 470 ms or QRS interval > 120 ms; had serum albumin < 2.5 g/dL; had platelets < 60,000/μL; had hemoglobin < 8 g/dL.

### Glasdegib pharmacokinetic and protein binding analyses

Plasma PK samples were analyzed for glasdegib concentrations at Covance Bioanalytical Services (Shanghai, China) using a validated, sensitive, and specific high-performance liquid chromatography–tandem mass spectrometry (HPLC–MS/MS) method. Plasma samples were stored at –70 °C until analysis and assayed within the 575 days of established stability. Calibration standard responses were linear over the range of 3–3000 ng/mL using a weighted (l/concentration^2^) linear regression. The lower limit of quantification (LLQ) for glasdegib was 3 ng/mL.

Assay accuracy, expressed as the inter-assay percentage relative error (%RE) of the mean glasdegib quality control (QC) sample concentrations ranged from –1.7% to 3.0% for the low (9 ng/mL), mid (100 ng/mL), and high (2250 ng/mL) QC samples. Assay precision, expressed as the inter-assay percentage coefficient of variation (%CV) of the mean glasdegib QC sample concentrations, was ≤ 6.0% across the low, medium, and high concentrations.

Plasma samples to measure the fraction of glasdegib not bound to human plasma proteins underwent equilibrium dialysis with phosphate-buffered saline (PBS). Dialyzed plasma and dialyzed PBS were diluted with non-dialyzed PBS and non-dialyzed plasma, respectively, to generate plasma:PBS mixed matrix samples that were analyzed using a validated, sensitive, and specific HPLC–MS/MS method to measure glasdegib concentrations. The fraction of glasdegib not bound to plasma proteins was calculated as the ratio of the glasdegib concentration in dialyzed PBS to the sum of glasdegib concentrations in dialyzed plasma and dialyzed PBS. Plasma samples were stored at –70 °C and dialyzed within the 575 days of established stability in plasma. Post-dialysis plasma:PBS samples were stored at –20 °C and assayed within the 63 days of established stability in plasma:PBS. Calibration standard responses were linear over the range of 1–1000 ng/mL using a weighted (l/concentration^2^) linear regression. The LLQ for glasdegib was 1 ng/mL.

Assay accuracy, expressed as the inter-assay  %RE of the mean glasdegib QC sample concentrations, ranged from –0.8% to 2.0% for the low (3 ng/mL), low–mid (40 ng/mL), mid (400 ng/mL), and high (800 ng/mL) QC samples. Assay precision, expressed as the inter-assay  %CV of the mean glasdegib QC sample concentrations, was ≤ 3.6% across the low, low–mid, mid, and high concentrations.

### Endpoints and statistical analyses

The primary objective of the study was to estimate the effect of renal impairment on the PK of a single, oral, 100-mg dose of glasdegib under fasted conditions. The PK concentration population was defined as all participants enrolled and treated who had ≥ 1 concentration. The PK parameter analysis population was defined as all participants enrolled and treated who had ≥ 1 of the PK parameters of primary interest, which were the PK parameters selected as primary endpoints. Using non-compartmental analysis of plasma concentration–time data, PK parameters assessed were: AUC from time 0 to the time of the last quantifiable concentration (AUC_last_); unbound AUC_last_; AUC_∞_; unbound AUC_∞_; CL/F; unbound CL/F; C_max_; unbound C_max_; fraction of unbound drug in plasma; time to C_max_ (t_max_); apparent volume of distribution of total drug (V_z_/F); unbound V_z_/F; and half-life (t_½_).

All participants who received ≥ 1 dose of study medication were included in the safety analyses and listings. As a secondary objective, the safety and tolerability of glasdegib were assessed by AE monitoring and changes in clinical laboratory results, ECGs, and physical examination findings. AEs were graded according to the Medical Dictionary for Regulatory Activities version 21.0.

A total of approximately 18 participants (approximately six per group) were to be enrolled into the study, based on EMA recommendations [14]. Glasdegib exposure in normal participants was the reference treatment. One-way analysis of variance was used to compare the natural log-transformed AUC_∞_ and C_max_ for each of the renal impairment groups (Test) with the normal group (Reference). Estimates of the adjusted mean differences (Test–Reference) and corresponding 90% confidence intervals (CIs) were obtained from the model. The adjusted mean differences and 90% CIs for the differences were exponentiated to provide estimates of the ratio of adjusted geometric means (Test/Reference) and 90% CIs for the ratios. Linear regression was used to analyze the potential relationship between appropriate PK parameters (CL/F or unbound CL/F) and renal function (CrCl and eGFR). Estimates of the slope and intercept, together with their precision (90% CI) and the coefficient of determination were obtained from the model. All other data were summarized descriptively, unless otherwise stated. Analyses were performed using SAS version 9.4.

A separate post hoc analysis of efficacy and safety was performed on patients from the BRIGHT 1003 MDS & AML clinical study (Phase Ib and II). All patients received glasdegib 100-mg QD plus low-dose cytarabine 20-mg BID subcutaneously [15, 16]. Patients were assigned as having normal renal function or mild or moderate renal impairment per the National Kidney Foundation, Kidney Disease Quality Outcomes Initiative criteria.

## Results

### Participants

A total of 18 participants were assigned and received glasdegib treatment between May 17, 2018 and September 19, 2018 at two centers in the United States. Six participants were assigned to each of the three groups. All participants completed the treatment and were included in the PK analysis. 11/18 participants were male and the majority were white (88.9%). Mean (range) age was 64.6 years (52–75) (Table 1). Mean (range) weight and BMI were 85.3 kg (60–104) and 30.2 kg/m^2^ (22–36), respectively. Thirteen participants received concomitant drug treatments during the study, the most frequent of which were acetylsalicylic acid and amlodipine (*n* = 6 each). Administration of concomitant medications that could impact the PK of glasdegib was not allowed during the conduct of the study.

**Table 1:** Demographic characteristics

Parameter, *n* (%)	Normal renal function group (*N *= 6)	Moderate renal impairment group (*N *= 6)	Severe renal impairment group (*N *= 6)	Healthy participants (*N* = 18)
Male	4 (66.7)	2 (33.3)	5 (83.3)	11 (61.1)
Age, years				
45–64	4 (66.7)	0	3 (50.0)	7 (38.9)
≥ 65	2 (33.3)	6 (100.0)	3 (50.0)	11 (61.1)
Mean (SD)	61.83 (4.07)	68.50 (3.27)	63.50 (8.53)	64.61 (6.16)
Range	57–67	66–75	52–71	52–75
Race				
White	5 (83.3)	5 (83.3)	6 (100.0)	16 (88.9)
Black or African American	1 (16.7)	1 (16.7)	0	2 (11.1)
Weight, kg				
Mean (SD)	83.1 (8.24)	87.0 (15.60)	85.7 (5.33)	85.3 (10.14)
Range	74–94	60–104	80–93	60–104
BMI, kg/m^2^				
Mean (SD)	28.7 (3.19)	31.4 (5.68)	30.6 (1.07)	30.2 (3.77)
Range	24–34	22–36	29–32	22–36

BMI was calculated as weight/(height × 0.01)^2^

*BMI* body mass index, *SD* standard deviation

### PK—total plasma glasdegib

**Fig. 1:**
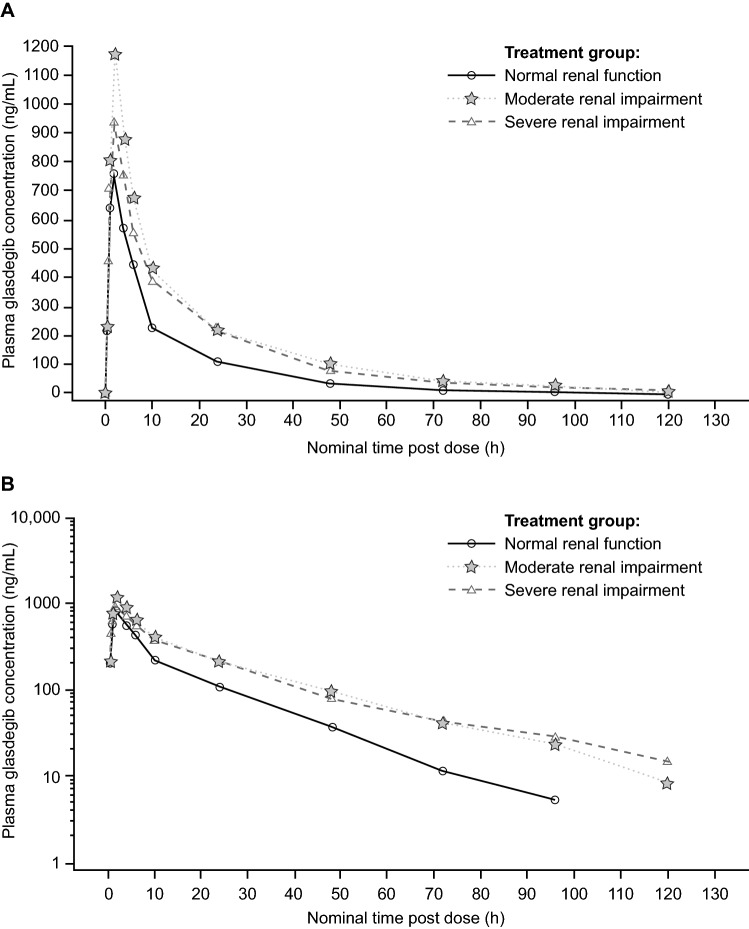
**a** Linear and **b** semi-logarithmic scales of median plasma glasdegib concentration–time profiles following a single 100-mg oral dose. The lower limit of quantification was 3 ng/mL

The median plasma glasdegib concentration–time profiles across renal function groups are shown in Fig. 1. Following a single, oral, 100-mg dose of glasdegib, median t_max_ was 2.0 h in both impairment groups and 1.5 h in the normal group, with the same range in all groups (1.0–2.0 h) (Table 2). Longer t_½_ mean values were observed in participants with moderate (22.7 h) and severe (25.0 h) renal impairment compared with those with normal renal function (16.8 h).

**Table 2:** Descriptive summary of plasma glasdegib PK parameters following a single 100-mg oral dose

Parameter^a^	Normal renal function group	Moderate renal impairment group	Severe renal impairment group
*n*/*N*	6/6	6/6	5/6
AUC_∞_, ng·h/mL	9599 (37)	19,660 (35)	19,430 (23)
Unbound AUC_∞_, ng·h/mL	719.2 (19)	1414 (42)	1430 (21)
AUC_last_, ng·h/mL	9477 (38)	19,180 (33)	16,620 (38)
Unbound AUC_last_, ng·h/mL	709.6 (19)	1380 (40)	1212 (39)
CL/F, L/h	10.43 (37)	5.093 (35)	5.148 (23)
Unbound CL/F, L/h	138.9 (19)	70.75 (42)	69.91 (21)
C_max_, ng/mL	791.2 (39)	1082 (28)	950.1 (50)
Unbound C_max_, ng/mL	59.25 (25)	77.83 (42)	69.25 (46)
f_u_, %	7.490 (19)	7.194 (20)	7.284 (12)
t_max_, h	1.50 (1.00–2.00)	2.00 (1.00–2.00)	2.00 (1.00–2.00)
V_z_/F, L	249.3 (31)	165.1 (20)	181.3 (42)
Unbound V_z_/F, L	3327 (18)	2295 (27)	2462 (36)
t_½_, h	16.83 (3.1923)	22.72 (3.7339)	24.98 (5.9889)

*AUC* area under the plasma concentration–time curve, *AUC*_*∞*_ AUC from time 0 to infinity, *AUC*_*last*_ AUC from time 0 to the time of the last quantifiable concentration, *CL/F* apparent clearance of total drug from plasma, *C*_*max*_ maximum plasma concentration,  *%CV* percentage coefficient of variation, *f*_*u*_ fraction of unbound drug in plasma, *N* number of participants in the treatment group and contributing to the summary statistics, *n* number of participants with reportable AUC_inf_, AUC_inf,u_, CL/F, CL_u_/F, V_z_/F, V_z,u_/F and t_½_, *PK* pharmacokinetics, *SD* standard deviation, *t*_*½*_ half-life, *t*_*max*_ time to C_max_, *V*_*z*_*/F* apparent volume of distribution of total drug

^a^Geometric mean (geometric  %CV) reported for all except median (range) for t_max_ and arithmetic mean (SD) for t_½_

**Fig. 2:**
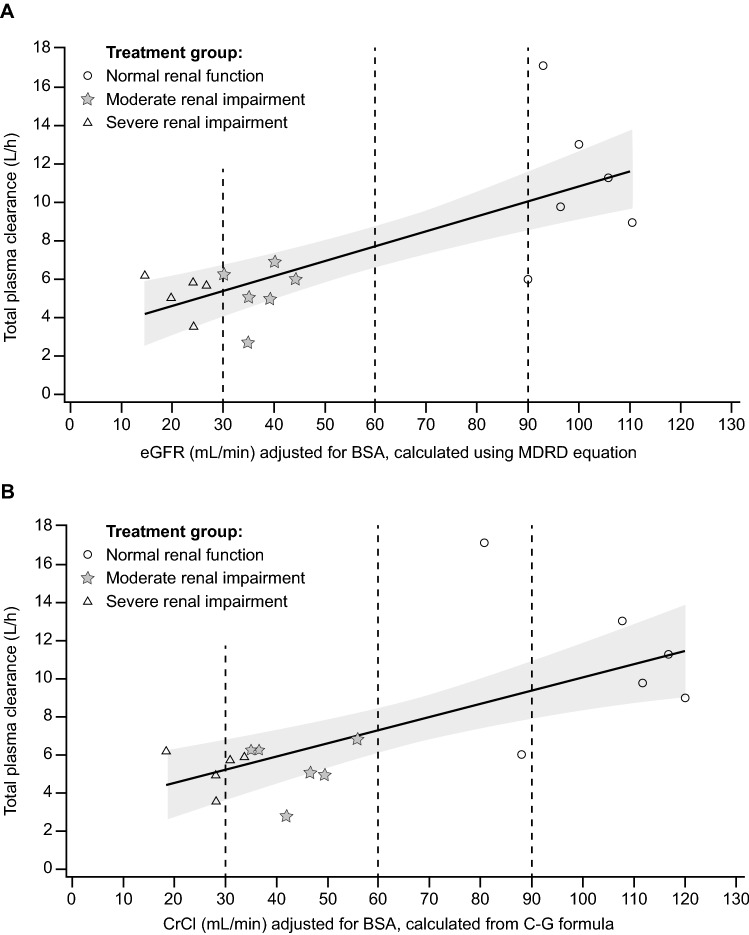
Regression plots of plasma glasdegib CL/F versus **a** eGFR and **b** CrCl following a single 100-mg oral dose. The solid line is the predicted line of the response variable. The shadow area is 90% confidence region for the response variable. Treatment group indicates the degree of renal impairment. **a** Day –1 eGFR calculated from MDRD equation is used in this presentation. Cl = 3.06 + 0.08 × eGFR; R = 0.5524835197; slope *p* value = 0.0006. **b** Day –1 CrCl calculated from C-G formula is used in this presentation. Cl = 3.09 + 0.07 × CrCl; R = 0.4550467229; slope *p* value = 0.0030. *BSA* body surface area, *C-G* Cockcroft–Gault, *Cl* clearance, *CL/F* apparent clearance of total drug from plasma, *CrCl* creatinine clearance, *eGFR* estimated glomerular filtration rate, *MDRD* modification of diet in renal disease

In a regression analysis using both CrCl and eGFR, a decrease in CL/F was observed with decreasing renal function (Fig. 2). Glasdegib mean oral clearance (CL/F) values decreased by approximately 50.0% in both renal impairment groups compared with the normal group: 5.1 L/h and 5.2 L/h for the moderate and severe renal impairment groups, respectively, compared with 10.4 L/h for the normal renal function group.

Glasdegib plasma exposure was approximately twofold higher in participants with moderate and severe renal impairment compared with participants with normal renal function: adjusted geometric mean AUC_∞_ ratios (90% CI) for renal impairment versus normal function were 204.8% (142.3–294.7%) and 202.4% (145.9–280.9%) for participants with moderate and severe renal impairment, respectively (Table 3). Mean C_max_ values were 1.4-fold and 1.2-fold higher for participants with moderate and severe renal impairment, respectively, compared with those with normal renal function: adjusted geometric mean C_max_ ratios (90% CI) for renal impairment versus normal function were 136.8% (96.7–193.3%) and 120.1% (76.9–187.6%) for participants with moderate and severe renal impairment, respectively. Variability in glasdegib exposure based on geometric  %CV ranged from 23% to 37% for AUC_∞_ and 28% to 50% for C_max_.

**Table 3:** Statistical summary of renal function group comparisons for total and unbound plasma glasdegib PK parameters

Parameter, units	Renal function comparison	Total plasma				Unbound plasma			
		Adjusted geometric means		Ratio (%) (test/reference) of adjusted means	90% CI (%) for ratio	Adjusted geometric means		Ratio (%) (test/reference) of adjusted means	90% CI (%) for ratio
		Test	Reference			Test	Reference		
AUC_∞_, ng·h/mL	Moderate vs normal	19,660	9599	204.79	(142.33–294.67)	1414	719.2	196.62	(139.81–276.54)
	Severe vs normal	19,430	9599	202.44	(145.87–280.93)	1430	719.2	198.83	(159.48–247.88)
AUC_last_, ng·h/mL	Moderate vs normal	19,180	9477	202.35	(141.32–289.74)	1380	709.6	194.54	(139.82–270.66)
	Severe vs normal	16,620	9477	175.35	(119.48–257.33)	1212	709.6	170.82	(123.50–236.27)
C_max_, ng/mL	Moderate vs normal	1082	791.2	136.76	(96.73–193.34)	77.83	59.25	131.35	(92.05–187.43)
	Severe vs normal	950.1	791.2	120.09	(76.86–187.63)	69.25	59.25	116.87	(79.87–170.99)
f_u_, %	Moderate vs normal	–	–	–	–	7.194	7.49	96.06	(78.53–117.49)
	Severe vs normal	–	–	–	–	7.284	7.49	97.25	(82.10–115.20)

*AUC* area under the plasma concentration–time curve, *AUC*_∞_ AUC from time 0 to infinity, *AUC*_*last*_ AUC from time 0 to the time of the last quantifiable concentration, *CI* confidence interval, *C*_*max*_ maximum plasma concentration, *f*_*u*_ fraction of unbound drug in plasma, *PK* pharmacokinetics

### PK – unbound plasma glasdegib

Glasdegib plasma protein binding was > 90.0% and the plasma-free fraction of glasdegib was not altered by renal impairment. Glasdegib protein binding was not saturable at the exposure range tested in this study, and in line with the observation that the protein binding in vitro was independent of glasdegib concentration over the tested range of 1–10 µM (1 µM = 375 ng/mL). The post-dose protein binding samples collected at 1 and 2 h post dose (expected t_max_ of glasdegib; see Table 2) were similar within each participant. The mean of the two time points within each participant was used to calculate individual unbound PK parameters. The geometric means for the fraction of unbound drug in plasma values were similar between groups: 7.5%, 7.2%, and 7.3% for normal, moderate, and severe groups, respectively (Table 3). Variability in unbound glasdegib exposure based on geometric  %CV ranged from 25% to 46% for unbound C_max_ and 19% to 42% for unbound AUC_∞_.

### BRIGHT 1003 MDS & AML clinical study

**Fig. 3:**
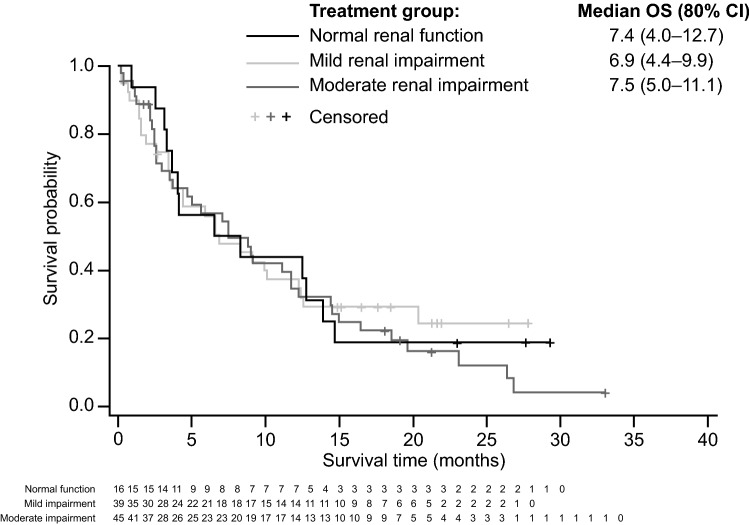
Overall survival by renal impairment and normal renal function in patients with AML or high-risk MDS in BRIGHT MDS & AML 1003. *AML *acute myeloid leukemia, *CI *confidence interval, *MDS *myelodysplastic syndrome, *OS *overall survival

Of 100 patients (*n* = 16 in Phase Ib; *n* = 84 in Phase II) with AML and MDS in BRIGHT 1003 MDS & AML, 39 and 45 had mild and moderate renal impairment, respectively, and 16 had normal renal function. The AE profiles were similar between patients with mild and moderate renal impairment and patients with normal renal function (Supplementary Table 1). Additionally, median overall survival (80% CI) was 6.9 (4.4–9.9) months for the mild renal impairment group, 7.5 (5.0–11.1) months for the moderate renal impairment group, and 7.4 (4.0–12.7) months for the normal renal function group (Fig. 3).

### Safety

Glasdegib was well tolerated, with five all-causality treatment-emergent AEs (TEAEs) reported in three participants. There were two TEAEs reported by one participant with severe renal impairment (infusion-site phlebitis and reversible airways obstruction), both of which were not considered to be treatment-related by the investigator. TEAEs of constipation and tremor were reported in one participant with moderate renal impairment, of which tremor was considered to be treatment-related by the investigator. One TEAE of musculoskeletal pain was reported by a participant with normal renal function, and was considered by the investigator to be treatment-related. All TEAEs were considered mild in severity and resolved by the end of the study.

No participants discontinued from the study. There were no deaths, serious AEs, severe AEs, or medical errors reported in this study. The most frequently observed laboratory abnormalities (abnormal baseline) were: blood urea nitrogen > 1.3 × upper limit of normal in one participant with moderate renal impairment and two participants with severe renal impairment; urine protein ≥ 1 in one participant with moderate renal impairment and two participants with severe renal impairment; and leukocyte esterase ≥ 1 in one participant with normal renal function and two participants with severe renal impairment. None of the laboratory test abnormalities, or changes in vital signs or ECG values were considered clinically significant by the investigator.

## Discussion

In the current study, and following a single, oral, 100-mg dose of glasdegib, plasma exposures, as measured by geometric mean AUC_∞_ values, were approximately twofold higher in participants with moderate and severe renal impairment compared with participants with normal renal function, with similar exposure observed between participants with moderate and severe renal impairment. Longer t_½_ mean values were observed in participants with moderate and severe renal impairment compared with those with normal renal function. This was probably due to a longer elimination phase in participants with renal impairment—a trend of decreased CL/F with decreasing renal function was also observed. A single oral dose of glasdegib 100-mg QD was safe and well tolerated both in participants with renal impairment and in those with normal renal function, and there were no discontinuations or serious AEs during the study. Given that the participants were in good general health, aside from differences in renal function, no clinically meaningful changes in laboratory parameters or vital signs were observed between the renal impairment and normal renal function groups.

The current study data support the findings from a population PK analysis of pooled data from patients with hematologic cancer and advanced solid tumor across Phase I and Phase II studies [13]. In the pooled analyses, the median glasdegib CL/F estimate was similar between patients with normal renal function (6.5 L/h) and mild renal impairment (6.2 L/h); therefore, an increase in glasdegib exposure was not expected with mild renal impairment and was not assessed in the current study. The median glasdegib CL/F for patients with moderate renal impairment decreased by approximately 26% compared with those with normal renal function. The magnitude of this effect was not considered clinically meaningful, and no dose adjustment was recommended in patients with mild or moderate renal impairment. No recommendations were possible for severe renal impairment due to lack of patients with baseline severe renal impairment in the analysis [13].

While the current study results showed an increase in glasdegib exposure in both participants with moderate and severe renal impairment, these findings have to be considered in the context of the safety profile of glasdegib and its clinical dose. The approved clinical dose of glasdegib 100-mg QD is 25% of the MTD (400-mg QD), providing a fourfold safety margin with regard to glasdegib exposure. MTD was assessed based on 28-day continuous once-a-day dosing—the standard for oncology drugs. Additionally, the safety of glasdegib 100-mg QD in mild and moderate renal impairment has previously been evaluated in the BRIGHT 1003 MDS & AML clinical study in patients with AML and MDS [15, 16]. No imbalances were noted in the AE profiles between patients with mild and moderate renal impairment and patients with normal renal function, suggesting lack of an impact of renal impairment on the glasdegib safety profile. Additionally, a post hoc analysis showed that the efficacy was comparable between patients with renal impairment and those with normal renal function.

Glasdegib is a substrate of CYP3A4. In a drug–drug interaction (DDI) study, following a single 200-mg dose of glasdegib, concomitant treatment with the strong CYP3A4 inhibitor ketoconazole increased glasdegib plasma exposure and peak plasma concentration by 140% and 40%, respectively, thus providing an upper limit of fold change in glasdegib exposures [12]. Given the fourfold margin between clinical dose and the MTD, no dose reduction of glasdegib was required in the BRIGHT MDS & AML 1003 trial, where patients with AML or high-risk MDS received concomitant CYP3A4 inhibitors [15, 16]. In the current study, following a single 100-mg dose of glasdegib, increases in glasdegib plasma exposures and peak plasma concentration were, respectively, 105% and 37% for moderate and 102% and 20% for severe renal impairment compared with participants with normal renal function. As these increases in exposures were less than the increases observed in the DDI study, where no dose modification was required due to the wide safety margins, no reduction in starting dose was deemed necessary in patients with renal impairment.

The discrepancy in the estimate of CL/F (26%) for patients with moderate renal impairment in the population PK analysis and the observed decrease in CL/F (50%) in the current study, might suggest an underestimation of the decrease in oral clearance in the population PK model. This possibility was the reason for prospectively including a moderate renal impairment group in the current clinical study. The population PK analysis was conducted using data generated based on a clinical formulation that was shown to be bioequivalent to the commercial ICH formulation used in the renal impairment study [17]. Additionally, when CrCl criteria were applied post hoc to recategorize participants, the number of participants with severe renal impairment was reduced to three due to the other three participants being reclassified as having moderate renal impairment. This was expected due to the differences in the MDRD versus Cockcroft–Gault criteria; however, there was no impact on the conclusions of the study regardless of using the MDRD or the Cockcroft–Gault approach.

In summary, the twofold increase in AUC_∞_ for glasdegib 100 mg observed in this study corresponded with equivalent plasma exposures at the 200-mg dose, which is still 50% lower than the MTD for glasdegib. Given the lower clinical dose providing robust safety coverage, the established safety in patients with cancer who have mild and moderate renal impairment, and the similar change in glasdegib exposure in participants with moderate and severe renal impairment in the renal impairment study, this facilitated the recommendation of no starting-dose adjustment in mild, moderate, or severe renal impairment, which is the current United States prescribing information label language for glasdegib.

## Electronic supplementary material

Below is the link to the electronic supplementary material.

Supplementary material 1 (DOCX 36 kb)

## Data Availability

Upon request, and subject to certain criteria, conditions, and exceptions (see https://www.pfizer.com/science/clinical-trials/trial-data-and-results for more information), Pfizer will provide access to individual de-identified participant data from Pfizer-sponsored global interventional clinical studies conducted for medicines, vaccines, and medical devices (1) for indications that have been approved in the US and/or EU or (2) in programs that have been terminated (i.e., development for all indications has been discontinued). Pfizer will also consider requests for the protocol, data dictionary, and statistical analysis plan. Data may be requested from Pfizer trials 24 months after study completion. The de-identified participant data will be made available to researchers whose proposals meet the research criteria and other conditions, and for which an exception does not apply, via a secure portal. To gain access, data requestors must enter into a data access agreement with Pfizer.
