# Supplementary material for: Evaluation of the impact of renal impairment on the pharmacokinetics of glasdegib in otherwise healthy volunteers
Source: Cancer Chemother Pharmacol. 2021 Jan 3;87(2):241–50. doi: 10.1007/s00280-020-04207-9 (PMC7870595; doi:10.1007/s00280-020-04207-9)
Supplement: Supplementary file 1 — Supplementary material 1 (DOCX 36 kb) [file 280_2020_4207_MOESM1_ESM.docx]

**Supplementary Table 1** Overview of all-causality treatment-emergent adverse events by renal impairment status in BRIGHT MDS & AML 1003

| *N* (%) | Normal renal function | Mild renal  impairment | Moderate renal impairment |
| --- | --- | --- | --- |
| Number of patients | 16 | 39 | 45 |
| Number of TEAEs | 228 | 589 | 757 |
| Patients with TEAEs | 16 (100.0) | 39 (100.0) | 45 (100.0) |
| Patients with TE serious AEs | 11 (68.8) | 32 (82.1) | 35 (77.8) |
| Patients with grade 3 or 4 TEAEs | 15 (93.8) | 35 (89.7) | 39 (86.7) |
| Patients with grade 5 TEAEs | 7 (43.8) | 12 (30.8) | 11 (24.4) |
| Patients discontinued due to TEAEs | 7 (43.8) | 15 (38.5) | 16 (35.6) |
| Patients with glasdegib dose reduced due to TEAEs | 3 (18.8) | 4 (10.3) | 8 (17.8) |
| Patients with backbone chemotherapy dose reduced due to TEAEs | 4 (25.0) | 3 (7.7) | 6 (13.3) |
| Patients with glasdegib temporary discontinuation due to TEAEs | 9 (56.3) | 19 (48.7) | 24 (53.3) |
| Patients with backbone therapy temporary discontinuation due to TEAEs | 7 (43.8) | 15 (38.5) | 13 (28.9) |

*AE* adverse event, *MedDRA*, Medical Dictionary for Regulatory Activities, *TE* treatment-emergent, *TEAE* treatment-emergent adverse event

Except for the number of AEs, patients were counted only once per treatment in each row

MedDRA (v19.1) coding dictionary applied
